# A cross-sectional study on stool- and gastrointestinal-related outcomes of Mexican infants consuming different formulae

**DOI:** 10.1186/s12887-023-04426-y

**Published:** 2023-12-15

**Authors:** Carlijn M. Maasakkers, Jeske H.J. Hageman, Olivia Balcazar Muñoz, Tomás Gómez Tamayo, Andrés Blanco Montero, Luis Gerardo Garza Lara, Regina Flores-López, Miriam Contreras Fernández, Sofía Morán Ramos, Tim T. Lambers

**Affiliations:** 1 FrieslandCampina, Amersfoort, The Netherlands; 2 Clínica Pediátrica Pigüi, Hospital Español, Mexico City, Mexico; 3 Clínica VITALI, Guadalajara, Jalisco Mexico; 4 Unidad de Genómica de Poblaciones, Instituto Nacional de Medicina Genómica, Mexico City, Mexico; 5 Departamento de Alimentos y Biotecnología, Facultad de Química, UNAM, Mexico City, Mexico; 6 P.O. Box 238, Wageningen, 6700 AE The Netherlands

**Keywords:** Infant nutrition, Digestive symptoms, Fecal characteristics, Infant formula

## Abstract

**Background:**

Immaturities present at birth, such as in the gut microbiome and digestive, nervous, and immune system, resolve with time. Nevertheless, this may result in mild digestive symptoms early in life, particularly in formula-fed infants. Formula composition and processing may impact this discomfort. This study therefore aimed to assess stool characteristics and gastrointestinal symptoms of healthy infants fed different formulae.

**Methods:**

A multicenter, cross-sectional, observational trial was performed in Mexico between November 2019 and January 2022, where exclusively formula-fed infants (n = 342, aged 1–4 months) were studied in four groups based on their existing formula use. Feeding was continued per practice following label instructions. For 7 days, parents/caregivers were requested to record fecal characteristics, using the Amsterdam Infant Stool Scale, and rate gastrointestinal symptoms. Stool samples were collected to determine pH, dry matter content, and fecal calprotectin levels.

**Results:**

Most infants had a soft/formed stool consistency, although odds for hard stools were different between groups. Gastrointestinal symptom scores revealed significant differences for burping and diarrhea, while other symptoms did not differ between groups. No significant differences between groups were found for stool frequency, dry matter content, and fecal pH. Although calprotectin was within the expected healthy ranges, significant differences among groups were seen. Furthermore, calprotectin significantly correlated with the severity of the gastrointestinal symptoms burping, flatulence, abdominal distension, and diarrhea.

**Conclusions:**

Differences in stool characteristics and specific differences in gastrointestinal symptoms were observed between different formula brand users. This may potentially be explained by the different composition and processing of the formulae, although there are multiple factors that influence the assessed outcomes.

**Trial registration:**

The study was registered in the Netherlands Trial Registry (NL7805), linked to https://trialsearch.who.int/, on 11/06/2019.

**Supplementary Information:**

The online version contains supplementary material available at 10.1186/s12887-023-04426-y.

## Background

The gastrointestinal system of infants is still underdeveloped. For example, the production of pancreatic enzymes is still low and the small intestine is relatively short [1]. Therefore, the capacity of digestion and absorption of nutrients is lower in infants, compared to the adult situation [2]. Furthermore, the gut microbiome, nervous, and immune system of the gastrointestinal tract is in an immature state [3]. These immaturities resolve with time as the gastrointestinal tract matures. However, early in life these immaturities may contribute to the development of mild gastrointestinal symptoms including colic, regurgitation, diarrhea, and constipation, particularly in formula-fed infants [4, 5]. These symptoms could result in distress for the infant. Roughly 50% of the infants develop at least one of these symptoms, of which regurgitation, infantile colic, and functional constipation are the most common [5]. Diet is an important factor in developing these gastrointestinal complications [6]. This is one of the many reasons why it is universally accepted that the optimal nutrition for a newborn infant is breastmilk. When a mother is unable to breastfeed her infant, infant formulae are the only food products that fulfill the nutritional requirements of infants during the first months of life [7]. With respect to formula-fed infants, particularly the protein source [8–10] and the optional ingredients pre- and probiotics [11, 12] have been considered as factors influencing these symptoms. It has been shown that formula-fed infants have a higher excretion of proteins compared to breastfed infants, which is caused by a reduced efficacy of protein absorption from the intestinal lumen [13, 14]. Besides general compositional differences between human milk and infant formula, such as protein, lipid, and carbohydrate complexity, the decreased absorption of infant formula is likely related to the processing of infant formulae [1, 15]. Predominantly heating during the processing of infant formulae can impact protein digestion [15]. Variations in formula composition and processing may thus impact gastrointestinal symptoms in infants. This study therefore aimed to assess the performance of four different formulae, that varied in composition and processing, with regard to stool- and gastrointestinal-related outcomes in a real-life setting. It was hypothesized that the different formulae would result in different stool- and gastrointestinal-related outcomes.

## Methods

### Study design

A multicenter, cross-sectional, observational study was performed, in which exclusively formula-fed infants were studied in four different groups based on their existing use of infant formula (IF A (FrieslandCampina, Amersfoort, The Netherlands), IF B, IF C, and IF D). The four different IFs were selected based on differences in composition and processing, the latter as illustrated by different blocked-lysine levels (shown in Additional Table 1), that were analyzed as indicators for protein glycation (as a result of the Maillard reaction) [16]. Feeding was continued per practice following the label instructions. Additional Table 1 displays the composition of the IFs, as presented on the labels, and from analyses.

This study was approved by the Ethical Committee of Hospital SMIQ S de R.L. de C.V. (CONBIOÉTICA-22-CEI-003-20171130, #16CI22014059) and by the Ethical Committee of Sociedad de Beneficencia Española, l.A.P. (CONBIOÉTICA-09-CEI-009-20170421, #15CI09016029) and was performed in accordance with the Declaration of Helsinki and its later amendments [17]. The study has been registered in the Netherlands Trial Registry, NL7805, on 11/06/2019 and was registered and approved by COFEPRIS (193300410D0017/2019) in Mexico.

### Participants

In total 364 subjects were enrolled between November 2019 and January 2022 by four different sites in Mexico; Clínica Pediátrica Pigüi and Hospital Star Médica Hip in Mexico City, Clínica VITALI in Guadalajara, and Hospital Christus Muguerza Sur in Monterrey. Healthy, term infants (gestational age 37–42 weeks, birth weight 2.5-4 kg), aged 1–4 months, were included. They were exclusively fed with a commercially available IF of interest for at least 3 weeks prior to inclusion in the study. For an infant to be included, parents/caregivers of the subjects had to agree to offer no weaning foods over the course of the study, had to be 18 years or older, should be fluent in Spanish, and own a smartphone with internet access. Subjects were excluded from the study if they: (I) had a birth weight-for-length (WFL) percentile > 85 or < 5, (II) had a congenital condition and/or previous or current illness that could interfere with the study as judged by the healthcare provider, (III) had a known or increased risk of cow’s milk allergy and/or lactose intolerance (i.e. one of the biological parents/caregivers and or siblings diagnosed with cow’s milk allergy, asthma, fever, etc.), (IV) were participating in another survey or trial, (V) had parents/caregivers that seemed unable or unwilling to comply with the protocol requirements, (VI) used antibiotics and/or other medication that influences the study outcomes as judged by the healthcare provider, as well as probiotic and/or prebiotic supplements (other than those found in the formula itself), at the time of screening and/or two weeks prior to the start of the study.

### Study procedures and questionnaires

After the study was properly explained, parents/caregivers were asked to sign the informed consent form. Subsequently, a screening visit took place in which anthropometric measurements were performed, and screening questionnaires were conducted to check the in- and exclusion criteria. Once enrolled in the study, the parents/caregivers received instructions on how to access and use the app which was used to fill in the questionnaires. Parents/caregivers were able to login with their individual account and see a listing of the task per day. They could open each of the questionnaires to insert the corresponding data. The app would prompt parents/caregivers to complete the required activities on a daily basis. Furthermore, stool sample kits were provided. To check the type of IF that was consumed, the parents/caregivers were requested to upload a picture of the IF used for feeding into the app as well.

Parents/caregivers were requested to complete daily diaries, to record time and description of each bowel movement, according to the Amsterdam Infant Stool Scale [18], for one week. This entailed the characterization of each stool on consistency (watery, soft, formed, hard), amount (four categories), and color (six categories). The primary endpoint of this study was stool consistency of the first stool per day for 7 days, where the categories soft and formed were grouped together as beneficial, compared to the categories watery and hard. Furthermore, stool frequency was extracted from these diaries based on the number of entries. These diaries were split in daily tasks to minimize recall bias.

Parents/caregivers were also asked to rate certain gastrointestinal symptoms in the daily diaries (score per day absent = 0 – very severe = 5), including abdominal distension, burping, flatulence, diarrhea, constipation, colic, diaper dermatitis, arching of the back, vomiting, and spit ups/regurgitation.

On day 7 parents/caregivers were requested to fill in the IGSQ (Infant Gastrointestinal Symptoms Questionnaire). The score was computed by summing the scores of all 13 items (range 13–65) [19].

### Stool sampling and analyses

Parents/caregivers were asked to collect stool samples from their child’s diaper on three different days, place these samples in a sterile plastic container, keep them at -20 °C, and bring them to the last visit on day 8. At the site, samples were stored at -20 °C and transported to the laboratory where they were stored at -80 °C until further analysis. Fecal samples were thawed at room temperature and homogenized for analyses. Standard pH strips (Merck-Milipore) were used to measure pH of diluted (1:10, sterile water) samples. Dry matter content was measured by lyophilizing a 100–200 mg aliquot for 24 h and weighing it. Levels of fecal calprotectin, an inflammatory marker [20, 21], were determined with a calprotectin ELISA kit (EK-CAL; Bühlmann Laboratories AG) according to the manufacturer’s instructions.

### Statistical analysis

A sample size of 100 subjects per group was based on a similar study comparing formulae on stool characteristics [22]. Accounting for a 10% drop-out rate this resulted in 440 subjects to be recruited. For continuous variables, means with standard deviations or medians with interquartile ranges were calculated, while for categorical variables percentages were computed. Stool consistency and stool color were analyzed with General Estimating Equation (GEE) applying multinomial logistic regression with repeated measurements, including all study days and IF groups. For stool amount and gastrointestinal symptoms, a similar GEE model but with ordinal multinomial accumulated responses was applied. If the variable IF type had an influence on the outcome measures (i.e. was significant in the GEE model) pair-wise post-hoc product comparisons were performed using Bonferroni adjustments.

Data normality of continuous variables was assessed with the Shapiro-Wilcoxon test. Since all the variables analyzed showed a nonparametric distribution, outliers were identified using 1.5xIQR. For comparison among IF groups of IGSQ scores, pH, dry matter content, and calprotectin levels, a Kruskal–Wallis test was used with Dunn’s post-hoc test to determine significant differences between groups, and Bonferroni adjusted p-values were used to correct for multiple comparisons. For IGSQ scores the Quade test was used as a nonparametric alternative to adjust for covariates. All analyses were performed unadjusted, and adjusted for sex (male/female), type of birth (C-section/natural), and age.

Intention to Treat (ITT) population entailed all included subjects. For the Per Protocol (PP) dataset all randomized subjects without protocol violations or deviations (e.g. out-of-window visit, prohibited medication use, or formula incompliance), and that had complete primary outcome data available were included. Due to the real-life setting of this study and the absence of large differences between ITT and PP populations, only ITT analyses are presented, with differences to PP indicated where applicable. Missing data were not imputed. Statistical analyses were performed in SPSS, SAS, and R, with p-values < 0.05 being considered statistically significant.

## Results

### Baseline characteristics and demographics

Of the 367 subjects screened, 364 were enrolled in the study, see flow diagram in Additional Fig. 1. In total 342 subjects completed the study; IF A: 102 subjects, IF B: 105 subjects, IF C: 100 subjects, and IF D: 35 subjects. The recruitment target of IF D was lowered (35 vs. 100) given the difficulties in recruitment due to a lower use of this product at the study locations in Mexico. In the PP analysis set, 296 subjects were taken along. Stool samples were available for 326 subjects, where 20 subjects were excluded due to protocol violations/deviations. Furthermore, for some subjects, not enough material was present for all analyses to be performed, therefore the objective measurements of pH, dry matter content, and calprotectin were conducted in 306, 302, and 304 samples respectively.

Table 1 displays the demographic characteristics of the four study groups. All groups contained slightly more boys than girls, ranging from 57 to 60%. The average age at enrollment was about 3 months. Small differences were found in weight and length.

**Table 1:** Demographics

	IF A	IF B	IF C	IF D
N enrolled	105	112	108	39
N completed	102	105	100	35
Sex (% male)	57.1%	60.7%	57.4%	59%
Age at enrolment (months)	3.0 ± 1.2	2.9 ± 1.1	3.2 ± 1.0	3.1 ± 1.1
Weight at enrolment (kg)^*^	5.5 ± 1.2^b^	5.7 ± 1.2^b^	6.2 ± 1.2^a^	5.7 ± 1.2^a,b^
Length at enrolment (cm)^*^	57.9 ± 4.5^b^	59.6 ± 4.4^a^	60.5 ± 4.4^a^	59.2 ± 4.6^a,b^
Head circumference at enrolment (cm)^#^	39.1 ± 2.2^b^	39.7 ± 2.0^a,b^	40.4 ± 2.1^a^	39.8 ± 4.0^a,b^
Mode of delivery (% vaginal)	34.3%	44.6%	49.1%	38.5%
Gestational age (weeks)^#^	38.6 ± 1.0^b^	39.0 ± 1.2^a^	38.9 ± 1.2^a,b^	38.9 ± 1.2^a,b^
Birth weight (kg)	3.1 ± 0.4	3.1 ± 0.4	3.1 ± 0.4	3.1 ± 0.5
Birth length (cm)	49.7 ± 2.2	50.2 ± 2.0	50.0 ± 2.6	49.0 ± 2.7
Twins/triplets (%)	10.5%	4.5%	0%	15.4%

Mean values ± SD are shown for continuous variables.^*^Indicates statistically significant ANOVA, ^*#*^*Indicates statistically significant Kruskal Wallis test. Superscript letters indicate significant differences by post hoc Bonferroni or Dunns test respectively, adjusted for multiple comparisons.*

### Stool characteristics

**Fig. 1:**
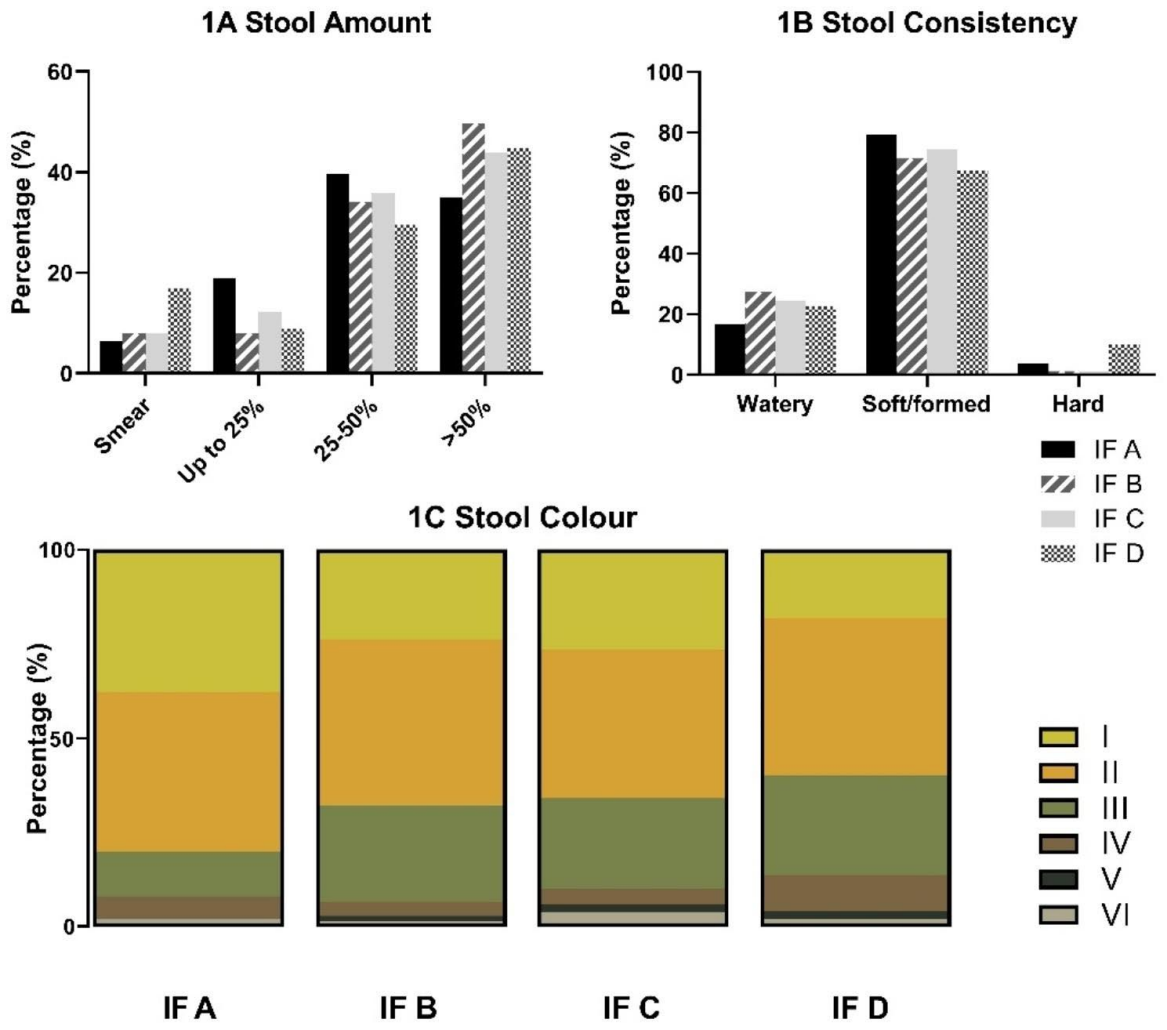
Stool characteristics. Figure shows the percentages of Stool amount (1**A**), Stool consistency (1**B**), Stool color (1**C**) of the first stool each day grouped per IF brand from the ITT population, according to the Amsterdam Infant Stool Scale.

No significant differences between groups were reported in stool frequency, dry matter content, or fecal pH (data not shown). Figure 1 shows the stool characteristics per formulae. For stool amount it was found that infants consuming IF A had lower odds of high amounts of stool compared to infants consuming IF B (OR 0.6 (95%CI 0.4–0.8)). Most stools were of a soft/formed consistency. Infants on IF D had a higher odds of hard stools vs. soft/formed compared to infants consuming IF B and IF C (OR 8.2 (95% CI 1.3–51.7) and OR 11.2 (95% CI 1.2-105.1) respectively). Infants consuming IF A had higher odds of having stool color I vs. III compared to the other IFs. All these differences remained significant after adjusting for sex, delivery mode, and age.

No differences were observed in general gastrointestinal symptoms between the four IFs, since the average IGSQ scores were similar for the four groups, between 23 and 24. For most individual gastrointestinal symptoms no differences between the four IFs were observed. Except for burping and flatulence, all gastrointestinal symptoms were reported to be absent in the majority of the registrations (percentages can be found in Additional Table 2). Infants consuming IF A had lower odds of more severe burping compared to infants consuming IF C (Table 2). Furthermore, infants consuming IF A had lower odds of having more severe diarrhea compared to the groups consuming IF B and IF D. These significances remained after adjustment with covariates, but in the PP analysis the difference between IF A and IF B on diarrhea was not found to be significance anymore. In the adjusted model the tendency of IF C having lower odds of more severe diarrhea compared to IF D became significant in both ITT and PP populations.

**Table 2:** Gastrointestinal symptom severity odd ratio’s

	IF A vs. IF B		IF A vs. IF C		IF A vs. IF D		IF B vs. IF C		IF B vs. IF D		IF C vs. IF D	
	OR (95% IC)	P-value	OR (95% IC)	P-value	OR (95% IC)	P-value	OR (95% IC)	P-value	OR (95% IC)	P-value	OR (95% IC)	P-value
Abdominal distention	1.25 (0.84;1.88)	1.00	1.63 (1.04;2.55)	0.20	1.18 (0.65;2.14)	1.00	1.30 (0.84;2.02)	1.00	0.94 (0.52;1.70)	1.00	0.72 (0.39;1.35)	1.00
Arching of the back	1.35 (0.83;2.22)	1.00	1.22 (0.75;1.98)	1.00	0.78 (0.40;1.50)	1.00	0.90 (0.54;1.50)	1.00	0.57 (0.29;1.13)	0.65	0.64 (0.33;1.25)	1.00
Burping	0.65 (0.43;0.96)	0.20	0.55 (0.37;0.83)	**0.03** ^*^	0.58 (0.31;1.06)	0.45	0.86 (0.58;1.27)	1.00	0.89 (0.49;1.62)	1.00	1.04 (0.57;1.90)	1.00
Colic	1.12 (0.73;1.71)	1.00	1.26 (0.80;1.97)	1.00	1.14 (0.62;2.09)	1.00	1.13 (0.73;1.73)	1.00	1.02 (0.57;1.84)	1.00	0.91 (0.50;1.66)	1.00
Constipation	1.20 (0.80;1.81)	1.00	1.74 (1.14;2.67)	0.06	0.87 (0.45;1.68)	1.00	1.45 (0.95;2.22)	0.50	0.72 (0.37;1.40)	1.00	0.50 (0.26;0.97)	0.24
Diaper dermatitis	0.98 (0.57;1.69)	1.00	1.18 (0.67;2.10)	1.00	0.76 (0.37;1.58)	1.00	1.20 (0.67;2.15)	1.00	0.77 (0.37;1.61)	1.00	0.64 (0.30;1.37)	1.00
Diarrhea	0.44 (0.25;0.76)	**0.02** ^*^	0.63 (0.36;1.12)	0.71	0.26 (0.12;0.54)	**0.002** ^*^	1.45 (0.85;2.48)	1.00	0.59 (0.29;1.20)	0.88	0.41 (0.20;0.84)	0.09
Flatulence	0.92 (0.60;1.39)	1.00	0.79 (0.52;1.20)	1.00	0.50 (0.29;0.88)	0.09	0.87 (0.58;1.30)	1.00	0.55 (0.32;0.95)	0.19	0.63 (0.37;1.10)	0.63

*Unadjusted odds ratio’s ITT population GEE model, WALD Type 3 test, p value with Bonferroni adjustment.* ^*^ *P < 0.05*

**Fig. 2:**
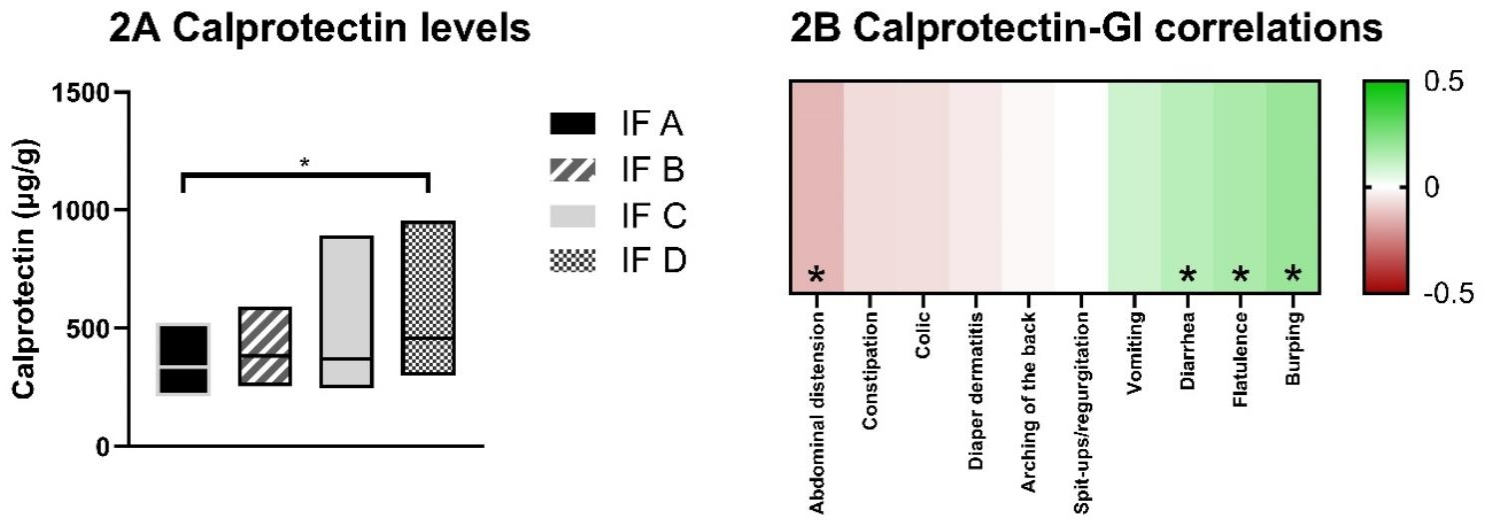
Calprotectin levels. 2**A **Median calprotectin levels with IQR per IF group. 2**B** Spearman’s rho correlation coefficients for calprotectin levels and gastrointestinal symptom severity scores. Green indicates a positive correlation meaning higher calprotectin levels correlated with a higher severity score, red indicates a negative correlation meaning higher calprotectin levels correlated with a lower severity score. *p < 0.05.

Gastrointestinal scores were also correlated to fecal calprotectin levels. More severe ratings of burping, flatulence, and diarrhea correlated significantly with higher calprotectin levels (Fig. 2B). Although calprotectin levels were in the expected range for healthy infants [23], levels were lower in fecal samples from infants on IF A compared to infants on IF D (Fig. 2A). This effect was not shown when outliers were excluded.

## Discussion

In this observational, real-life setting study in Mexico, gastrointestinal and digestion-related outcomes of different commercially available infant formulae were studied in healthy term subjects. Most infants had soft or formed stools, although odds for having hard stools were different between groups. Further significant differences were found in stool amount and color. No significant differences between groups were reported on stool frequency, dry matter content, or fecal pH. Gastrointestinal symptom scores revealed significant differences for burping and diarrhea, while other gastrointestinal symptoms did not differ. Interestingly, although calprotectin levels were within the expected ranges for healthy infants, differences among groups could be established. Furthermore, calprotectin levels correlated with the severity of the gastrointestinal symptoms burping, flatulence, abdominal distension, and diarrhea.

Several gastrointestinal disorders are associated with changes in stool characteristics, causing them to be monitored by parents and in clinical practice [18]. Odds for having hard stools were higher for infants on IF D compared to IF B and IF C, although differences in dry matter content were not found. This could relate to the limited sample size in the current study, since dry matter content (as determined by lyophilization) was different among the consistency categories, as similarly demonstrated previously in adults [24]. Breastfed infants generally have softer stools compared to formula-fed infants, with formula composition affecting factors such as fecal soap formation, that may explain variations among formula-fed infants [25]. Also fecal color from healthy infants varies, ranging from yellow to green, where breastfed infants more often have a yellow color compared to formula-fed infants [26]. The current study showed that infants consuming IF A had higher odds of having a yellow stool color compared to the other IFs. The physiological relevance of color variations within the healthy range is unknown, and clinically less relevant than discriminating category 5 and/or 6 from others. However, the current study thus suggests that fecal color may differ per the consumed formula type, similar as stool consistency.

Next to the stool characteristics, gastrointestinal symptoms were also studied. As the study population consisted of healthy infants, it was in line with expectations that many gastrointestinal symptoms were reported to be absent or very mild in many of the registrations. This is in line with observations of several countries where mild gastrointestinal disorders have previously been observed in otherwise healthy infants [4]. In this study, only burping and flatulence were reported more often. Due to the low prevalence of GI symptoms, the differences between study products were expected to be limited. Nonetheless, the current study revealed that infants consuming IF A had lower odds of more severe burping and diarrhea compared to infants consuming IF C and infants on IF B and IF D, respectively. Significant differences were thus found in otherwise healthy infants.

The inflammatory marker calprotectin was lower in fecal samples of infants consuming IF A compared to IF D. Interestingly, fecal calprotectin correlated with the gastrointestinal symptoms burping, flatulence, and diarrhea. With respect to gastrointestinal complications, fecal calprotectin has limited use in standard clinical practice [20]. Fecal calprotectin is, for example, only informative in the diagnosis and monitoring of inflammatory bowel disease and to distinguish it from functional gastrointestinal disorders, whereas application in the diagnosis of colic and constipation is limited [20]. Although clinical relevance will therefore be limited in healthy infants, the correlation of fecal calprotectin with the symptoms burping, flatulence, and diarrhea may highlight a potential mechanistic interaction which may be interesting for future studies.

Combined, the differences in gastrointestinal symptoms and fecal calprotectin might partly be related to the different formulae consumed, with diet impacting digestive challenges that may be caused by the immature gastrointestinal system in early life [4].

Different infant formulae are available on the market, with variation in composition, predominantly related to the protein and fat source, and the presence of optional ingredients. With respect to the protein source, hydrolyzed protein formulae have previously been demonstrated to reduce digestive complications mostly associated with regurgitation [8]. Furthermore, fat sources, i.e. bovine milk or plant oil-derived and modified plant oils, such as beta-palmitate, can impact gastrointestinal outcomes through their effect on fecal soap formation. This may impact clinical outcomes, such as bone strength, gut microbiome composition, and crying in infants [9, 10, 27, 28]. Pre- and probiotics are considered for the management of pediatric gastrointestinal disorders as well, as has been reviewed in detail by the ESPGHAN taskforce for pre- and probiotics [11, 12]. Recommendations for the use of specific probiotic strains were made by this taskforce for the management of functional abdominal pain disorders and infant colic, among others [12]. More recently, characteristics related to processing [15, 29] and casein mineralization [30] have also been suggested to impact digestion and gastrointestinal-related outcomes. For example, protein denaturation and glycation have been demonstrated to impact overall IF protein digestion [15, 31, 32] and may thus warrant further investigation. All formulae in this study contained non-hydrolyzed protein sources and three of the four formulae contained different oligosaccharides (IF A, C, D). Three IFs included a human milk oligosaccharide (IF A, B, D), and one was supplemented with a probiotic (IF B) (see Additional Table 1). Moreover, formulae varied in indicators of glycation, ranging from a blocked lysin percentage of 6.1% (IF A) to 18.5% (IF C). These levels are indicative for differences in heating applied during formula production and may impact digestion and absorption as indicated by both in vitro and in vivo studies [29, 33, 34]. Combined, the differences in formula composition (particularly related to optional ingredients such as oligosaccharides and probiotics) and processing might therefore have contributed to the differences seen in this study. Theoretically, this effect could be either direct and/or through alterations in the gut microbiome. However, a direct association between IF characteristics with clinical outcomes in this observational study is not possible, as assessed outcomes are multifactorial and can be affected by many factors.

Strengths of this study include the observational setting, which enables real-world data collection. Subjects were recruited in different centers across Mexico, making the results generalizable to the wider population of, at least, Mexican infants. A limitation of the study was that outcomes were assessed based on parental reports, likely increasing the variation. Therefore, several objective and potential mechanistic measures, and the validated Amsterdam Infant Stool Scale [18] were also included in the current study. Furthermore, the lower number of subjects in one of the four groups, although representative of the frequency of use, could have resulted in reduced statistical power to determine significant differences. Additionally, no breastfed reference was included. Lastly, due to the observational nature of the study and cross-sectional assessment of the outcome measures no longitudinal data was obtained meaning no causal conclusions can be drawn.

## Conclusion

Exclusively formula-fed infants, using different commercially available formulae, were compared in this study. Although only healthy subjects were included, differences in stool characteristics and gastrointestinal symptoms were observed. Despite the fact that assessed outcomes are multifactorial, variations may potentially be explained by the different compositions and processing conditions of the formulae. Interestingly, fecal calprotectin correlated with the severity of the gastrointestinal symptoms burping, flatulence, abdominal distention, and diarrhea which may be a subject for further studies.

### Electronic supplementary material

Below is the link to the electronic supplementary material.

Supplementary Material 1

Supplementary Material 2

Supplementary Material 3

## Data Availability

The authors and study sponsor encourage and support the responsible and ethical sharing of data from clinical trials. De-identified participant data from the final research dataset used in the published manuscript may only be shared under the terms of a Data Use Agreement. Requests may be directed to: tim.lambers@frieslandcampina.com.
