# Supplementary figures and images for: A cross-sectional study on stool- and gastrointestinal-related outcomes of Mexican infants consuming different formulae
Source: BMC Pediatr. 2023 Dec 15;23:634. doi: 10.1186/s12887-023-04426-y (PMC10722798; doi:10.1186/s12887-023-04426-y)

Additional Figure 1 - Flow Chart

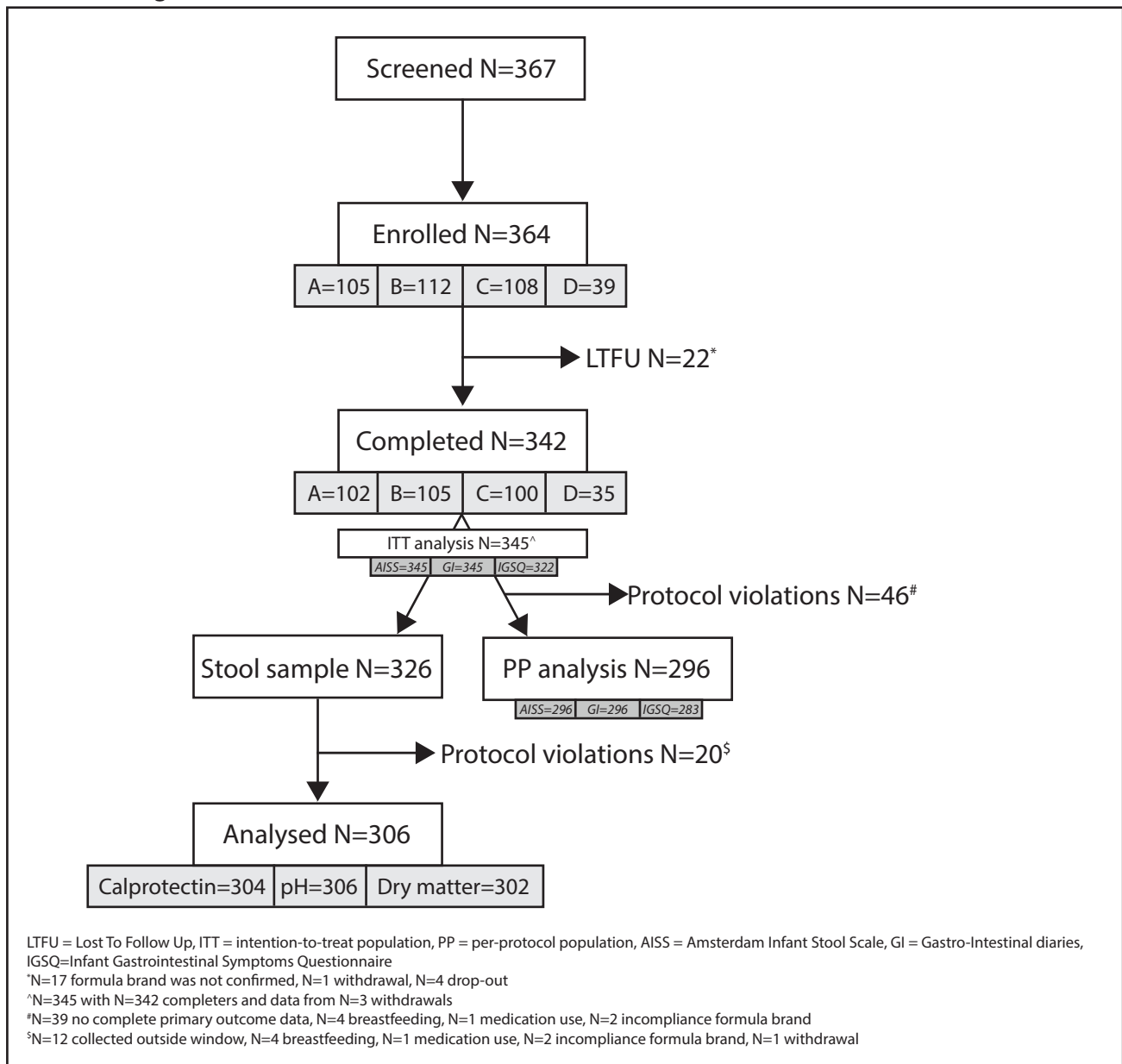

Supplement: Supplementary file 1 — Supplementary Material 1 [file 12887_2023_4426_MOESM1_ESM.pdf]
