# Supplementary material for: A cross-sectional study on stool- and gastrointestinal-related outcomes of Mexican infants consuming different formulae
Source: BMC Pediatr. 2023 Dec 15;23:634. doi: 10.1186/s12887-023-04426-y (PMC10722798; doi:10.1186/s12887-023-04426-y)
Supplement: Supplementary file 2 — Supplementary Material 2 [file 12887_2023_4426_MOESM2_ESM.docx]

*Additional Table 1 - Composition and characteristics of IFs used in this study*

| **Per 100g** | **IF A** | **IF B** | **IF C** | **IF D** |
| --- | --- | --- | --- | --- |
| Energy (kJ) | 2109 | 2170 | 2033 | 2147 |
| Macronutrients |  |  |  |  |
| *Protein (g)* | 11 | 9.6 | 9.2 | 10.6 |
| *Fat (g)* | 27 | 27.3 | 25 | 28.2 |
| *Carbohydrates (g)* | 54 | 58.6 | 56 | 54.3 |
| Optional ingredients |  |  |  |  |
| *Galacto-oligosaccharides (GOS) (g)* | 1.9 | - | 1.5 | - |
| *Fructo-oligosaccharides (FOS) (g)* | - | - | - | 1.6 |
| *Polydextrose (PDX) (g)* | - | - | 1.5 | - |
| *2’-fucosyllactose (2’-FL) (g)* | 0.19 | 0.19 | - | 0.16 |
| *Probiotics* | - | *Lactobacillus reuteri* | - | - |
| Indicators of glycation |  |  |  |  |
| *Furosine (mg)* | 30.1 | 69.2 | 83.6 | 42.0 |
| *Blocked lysin (%)* | 6.1 | 14.8 | 18.5 | 8.9 |

*All values are per 100 g powder as declared on label; exceptions are furosine and blocked lysin, which are analyzed values.*
