# Supplementary material for: A cross-sectional study on stool- and gastrointestinal-related outcomes of Mexican infants consuming different formulae
Source: BMC Pediatr. 2023 Dec 15;23:634. doi: 10.1186/s12887-023-04426-y (PMC10722798; doi:10.1186/s12887-023-04426-y)
Supplement: Supplementary file 3 — Supplementary Material 3 [file 12887_2023_4426_MOESM3_ESM.docx]

*Additional* *Table 2 - Percentages of all gastrointestinal symptom scores for the ITT population*

|  |  | IF A | IF B | IF C | IF D |  |
| --- | --- | --- | --- | --- | --- | --- |
| IGSQ total score (SD) |  | 23.9 ± 0.7 | 23.3 ± 0.7 | 23.1 ± 0.7 | 24.0 ± 0.7 |  |
| Abdominal distension | Absent | 59.0% | 65.8% | 70.9% | 64.6% |  |
|  | Very mild | 25.1% | 18.1% | 17.0% | 17.5% |  |
|  | Mild | 7.6% | 7.3% | 7.0% | 10.4% |  |
|  | moderate | 6.7% | 7.8% | 3.2% | 5.4% |  |
|  | Quite/very severe | 1.6% | 1.0% | 1.8% | 2.1% |  |
| Arching of the back | Absent | 69.4% | 73.2% | 70.0% | 60.0% |  |
|  | Very mild | 20.9% | 12.6% | 16.9% | 17.9% |  |
|  | Mild | 8.9% | 5.9% | 7.0% | 13.3% |  |
|  | Moderate/quite/very severe | 5.3% | 8.3% | 6.0% | 8.3% |  |
| Burping | Absent | 15.6% | 11.0% | 7.8% | 17.1% |  |
|  | Very mild | 36.0% | 27.3% | 27.5% | 22.1% |  |
|  | Mild | 21.6% | 27.5% | 25.3% | 22.1% |  |
|  | moderate | 24.6% | 31.1% | 32.9% | 37.5% |  |
|  | Quite/very severe | 2.1% | 3.1% | 5.0% | 4.2% |  |
| Colic | Absent | 52.2% | 60.5% | 63.6% | 61.7% |  |
|  | Very mild | 21.8% | 21.8% | 19.4% | 20.0% |  |
|  | Mild | 12.5% | 11.4% | 11.7% | 10.4% |  |
|  | moderate | 5.7% | 5.1% | 3.7% | 5.4% |  |
|  | Quite/very severe | 1.9% | 1.3% | 1.6% | 2.5% |  |
| Constipation | Absent | 57.6% | 63.1% | 70.6% | 59.6% |  |
|  | Very mild | 17.2% | 15.1% | 14.5% | 9.2% |  |
|  | Mild | 13.3% | 8.8% | 6.5% | 8.3% |  |
|  | moderate | 8.2% | 7.8% | 6.3% | 11.7% |  |
|  | Quite/very severe | 3.7% | 5.2% | 2.1% | 11.2% |  |
| Diaper dermatitis | Absent | 79.0% | 79.0% | 82.2% | 75.0% |  |
|  | Very mild | 14.0% | 13.3% | 9.5% | 12.1% |  |
|  | Mild | 5.2% | 4.0% | 4.6% | 8.8% |  |
|  | moderate | 1.6% | 3.3% | 1.6% | 3.3% |  |
|  | Quite/very severe | 0.3% | 0.6% | 2.1% | 0.8% |  |
| Diarrhea | Absent | 90.1% | 80.2% | 85.6% | 71.7% |  |
|  | Very mild | 7.2% | 10.5% | 7.0% | 10.0% |  |
|  | Mild | 1.6% | 4.8% | 3.8% | 10.0% |  |
|  | moderate | 1.0% | 3.5% | 2.2% | 5.4% |  |
|  | Quite/very severe | 0.1% | 1.0% | 1.3% | 2.9% |  |
| Flatulence | Absent | 14.9% | 17.1% | 12.2% | 7.9% | |
|  | Very mild | 31.2% | 23.6% | 26.3% | 20.0% | |
|  | Mild | 20.5% | 23.0% | 23.9% | 24.6% | |
|  | Moderate | 25.6% | 29.9% | 28.9% | 32.9% | |
|  | Quite severe | 1.6% | 1.0% | 1.2% | 2.5% | |
|  | Severe | 6.2% | 5.4% | 7.5% | 12.1% | |
